# Action of Salol on the Kidneys

**Published:** 1890-12-13

**Authors:** 


### THERAPEUTICS.

### ACTION OF SALOL ON THE KIDNEYS.

Some time ago we pointed out the fallacy of the claims put forward for salol as being free from the alleged dangers of salicylic acid, since being broken up by the action of the pancreatic juice into its components of salicylic acid and phenol it must possess the advantages and disadvantages of the acid, plus those incident to phenol. Dr. Hesselbach has recently investigated the question with great care, and finds that though after a dose of 30 grains the elimination of salicyluric acid by the kidneys commences within half an hour, it is not completed for several days, the dark greenish hue of the urine (carboluria, so-called) being observable. It follows that if the use of the drug be continued every few hours, elimination not keeping pace with injection, cumulative and poisonous effects may easily be produced. To which of the constituents of salol the occasional ill results are due has been much disputed, but Dr. Hesselbach is certain that phenol is, at any rate in some cases if not in most, the toxic agent. One which ended fatally is worth recording. A servant girl, aged 22, stout and somewhat anæmic, but in fairly good health, was attacked on May 18th with acute articular rheumatism, for which she was treated with salicylate of soda, to the great relief of the pain, except in the ankles. On June 8th she was given 120 grains of salol within eight hours, after which she became unconscious, and died on the 12th. Before this, on June 10th, no urine having been passed for two days, the catheter was used and 8½ oz. drawn off—pale yellow, slightly turbid, and containing traces of albumen, salicyluric acid, and phenol. A post-mortem examination showed recent acute nephritis, in which the changes in the epithelium were conspicuous. A series of experiments on rabbits undertaken by Dr. Hesselbach with toxic doses of phenol, salol, and salicylic acid showed that the changes in the kidney produced by the two former drugs were similar, while those caused by the acid alone were of a different character. Phenol led to anæmia of the kidney and acute fatty degeneration of the epithelium of the convoluted tubes, salicylic acid to hyperæmia of the kidney, and hæmorrhages into the intestinal tissue and tubules, with very little epithelial degeneration. Phenol acted primarily on the cortex, and salicylic acid on the medullary portion, the action of each extending to the other regions only when given in extremely large doses. After salol the post-mortem appearance presented by the kidney corresponded with those caused by phenol, the evidences of simultaneous salicylic acid poisoning being recognized only when the dosage was excessive. Phenol poisoning is far more likely to occur when the kidneys are already in any way diseased, and Dr. Küster has remarked the same susceptibility in febrile and anæmic patients, especially females. Salol, therefore, should always be administered with great caution, and entirely avoided when renal disease, acute or chronic, exists. Indeed, we fail to see any reason whatever for substituting this drug for salicylic acid, which can now be obtained in the artificial form absolutely free from cresotonic acid, and at a cost scarcely if at all, greater than hitherto.

